# Global 10 year ecological momentary assessment and mobile sensing study on tinnitus and environmental sounds

**DOI:** 10.1038/s41746-025-01551-z

**Published:** 2025-03-13

**Authors:** Robin Kraft, Berthold Langguth, Jorge Simoes, Manfred Reichert, Winfried Schlee, Rüdiger Pryss

**Affiliations:** 1 Institute of Medical Data Science, University Hospital Würzburg, Würzburg, Germany; 2 Institute of Clinical Epidemiology and Biometry, University of Würzburg, Würzburg, Germany; 3 Institute of Databases and Information Systems, Ulm University, Ulm, Germany; 4 Department of Clinical Psychology and Psychotherapy, Ulm University, Ulm, Germany; 5 Department of Psychiatry and Psychotherapy, University of Regensburg, Regensburg, Germany; 6 Department of Psychology, Health and Technology, University of Twente, Enschede, The Netherlands; 7 Institute of Information and Process Management, Eastern Switzerland University of Applied Sciences, Rapperswil, Switzerland

**Keywords:** Signs and symptoms, Neuroscience, Translational research

## Abstract

In most tinnitus patients, tinnitus can be masked by external sounds. However, evidence for the efficacy of sound-based treatments is scarce. To elucidate the effect of sounds on tinnitus under real-world conditions, we collected data through the TrackYourTinnitus mobile platform over a ten-year period using Ecological Momentary Assessment and Mobile Crowdsensing. Using this dataset, we analyzed 67,442 samples from 572 users. Depending on the effect of environmental sounds on tinnitus, we identified three groups (T-, T+, T0) using Growth Mixture Modeling (GMM). Moreover, we compared these groups with respect to demographic, clinical, and user characteristics. We found that external sound reduces tinnitus (T-) in about 20% of users, increases tinnitus (T+) in about 5%, and leaves tinnitus unaffected (T0) in about 75%. The three groups differed significantly with respect to age and hearing problems, suggesting that the effect of sound on tinnitus is a relevant criterion for clinical subtyping.

## Introduction

Tinnitus refers to the perception of sound in the absence of a corresponding external sound source^1^. Each patient with tinnitus requires comprehensive diagnostic assessment, as in rare cases, tinnitus can be the first symptom of a severe disorder such as vestibular schwannoma or vascular abnormalities^2,3^. In the majority of cases, tinnitus is a symptom of initial injury to the cochlea, such as noise trauma, as well as sudden or age-related hearing loss^3,4^. The relationship between environmental noise and tinnitus is complex. First, the risk for age-related hearing loss—and in consequence also the risk for tinnitus—is increased by long-term noise exposure^4,5^. Second, exposure to loud noise is frequently causing transient tinnitus^6^. Third, in many tinnitus patients, the loudness of the tinnitus can be reduced by the presence of external sounds^6–8^. This effect is called *tinnitus masking* and has been known for at least half a century^8,9^. If the intensity of the external sound is sufficient, the tinnitus can be made inaudible, i.e., it can be *masked*^6,10^. A distinction is made between *complete* and *partial* masking^3,11^. The masking effect may even persist for some time after removal of the masking sound^8^, which is referred to as *residual inhibition*^6,7^.

However, despite intensive efforts to use sound for the treatment of tinnitus, there is only very limited evidence for the efficacy of these treatment options^3,12–15^ and it is debated whether some forms of sound treatment may even cause harm in the long term^16^. Moreover, environmental sounds do not always result in a reduction of tinnitus perception. Some patients report that external sounds worsen their tinnitus while others have the impression that their tinnitus remains unaffected by external sounds^6,17^. The general hypothesis is that tinnitus patients differ in their responsiveness to environmental sounds. In this context, we aimed to investigate (1) whether the variable effect of environmental sounds on tinnitus can be confirmed by Ecological Momentary Assessment (EMA) under real-world conditions and (2) whether the reaction to environmental sounds represents a relevant criterion for patient subtyping.

**Fig. 1:**
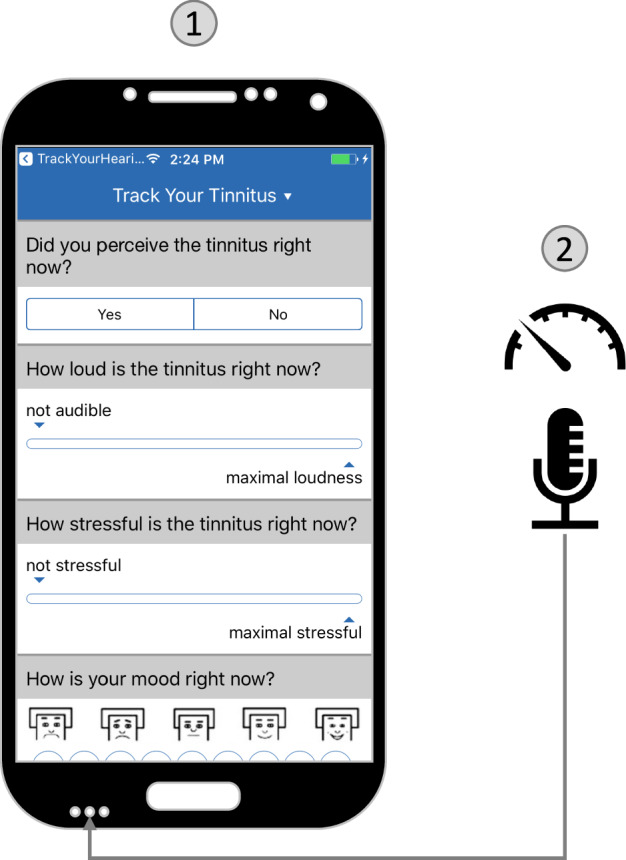
Data collection of the TrackYourTinnitus (TYT) mobile platform. Data are collected using ① a repeating EMA questionnaire completed by the user and ② an automatic recording of the environmental sound level via the device microphone.

To investigate the interrelationships between objective sound measurement and subjective tinnitus perception, we used the combination of EMA and mobile sensing, which has become an established method for collecting both subjective and objective health-related data^18–20^. This combination has already been used in various behavioral and mental eHealth application areas^21^. However, to our knowledge, the combined use of EMA and Mobile Crowdsensing (MCS) to investigate the relationship between environmental sounds and subjectively reported tinnitus symptoms is a novelty. MCS denotes a mobile sensing paradigm in which mobile devices of a community are leveraged to collectively assess phenomena of common interest^22^. The TrackYourTinnitus (TYT) platform is a mobile-based solution that uses EMA and MCS data collection strategies to capture fluctuations of individual tinnitus symptoms^23^. For this purpose, a repeating EMA questionnaire is used to assess individual tinnitus perception in real time, while environmental sound levels are recorded in parallel utilizing the device’s microphone, as illustrated in Fig. 1^24,25^.

Many preparatory steps had to be performed to enable a correct interpretation of the collected sound level sensor data by analyzing the dataset^25,26^ as well as the application code^25^ and cleaning the existing data in terms of outliers and sensor errors from a *data perspective*^27^. Based on these preparatory steps, we investigated the effect of environmental sound levels recorded with mobile devices on the perceived loudness of tinnitus.

## Results

The comparison of the subjective EMA data on perceived tinnitus loudness (TL) with the sensor-based objective measurements of environmental sound levels (SL) confirmed the hypothesis that tinnitus patients differ in their responsiveness to environmental sound.

### Identified patient subgroups

**Fig. 2:**
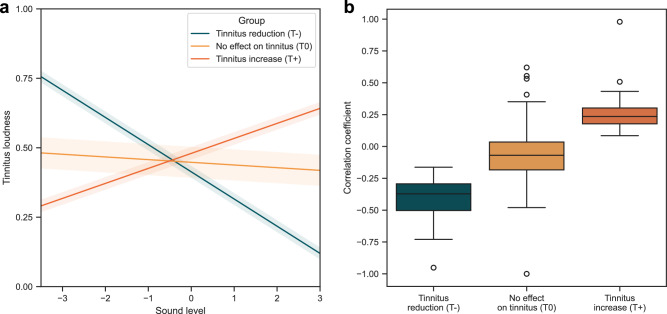
Illustration of the groups T-, T0, and T+. The three identified groups are shown in which increasing environmental sounds leaded to tinnitus reduction (*T*- = 18.2%), had no effect on tinnitus loudness (T0 = 77.1%), and led to tinnitus increase (T+ = 4.7%). **a** Estimated mean trajectories and variances per group. **b** Distribution of Pearson correlation coefficients between tinnitus loudness and environmental sound level for each individual patient per group.

We were able to differentiate three groups of patients, depending on the influence of environmental sounds on tinnitus: Patients in which the subjective tinnitus loudness decreased on average with increasing environmental sound level (T-), patients in which subjective tinnitus loudness increased on average with increasing environmental sound level (T+), and patients in which tinnitus loudness remained largely independent of the environmental sound level (T0). Of the 572 subjects studied, in 104 (18.2%) we observe a masking effect (T-), in 441 (77.1%) no effect (T0), and in 27 (4.7%) an increase in tinnitus loudness (T+) with increasing environmental sound levels. The parameter estimates for each of the three identified groups are shown in Table 1. The model results are illustrated in Fig. 2.

**Table 1:** Parameter estimates of the three identified groups of growth trajectories with respect to the relationship between environmental sound level and tinnitus loudness in the final growth model

	Group		
	Tinnitus reduction (T-)	No effect on tinnitus (T0)	Tinnitus increase (T+)
Intercept	0.413	0.448	0.480
Sound level	−0.098	−0.010	0.054
Intercept variance	0.020	0.055	0.021
Residual variance	0.030	0.030	0.030

### Group comparison

The comparison of the three groups (T-, T0, T+) with respect to clinical and demographic characteristics revealed significant differences in age, age at onset of tinnitus, and hearing problems, which indicates that the effect of sound on tinnitus is a relevant criterion for clinical subtyping. The average age was 50.9 (SD = 13.2) in the T- group, 56.9 (SD = 12.9) in the T0 group, and 57.5 (SD = 11.7) in the T+ group. However, for age at onset of tinnitus, when adjusting for the age with an ANCOVA, the effect is no longer significant (*F* = 0.307, *η*^2^ = 0.001, *p* = 0.736). Furthermore, there are non-significant indications of differences in gender and a question on subjective tinnitus maskability in the registration questionnaire. The proportion of women is 35.6% in T-, 26.5% in T0, and 29.6% in T+. The question “Is your tinnitus reduced by music or by certain types of environmental sounds such as the noise of a waterfall or the noise of running water when you are standing in the shower?” was answered with “yes” by 58.7% of users in the T- group, 44.7% in the T0 group, and 55.6% in the T+ group. The descriptive statistics for the total dataset and a comparison between the three groups are shown in Table 2. Post-hoc tests for these comparisons can be found in Supplementary Tables 1–3.

**Table 2:** Descriptive statistics for the total dataset used in the analysis and a comparison between the groups T-, T0, and T+

	Total	T-	T0	T+	Test Statistic (DoF)	Effect Size	*p*	*p*_*adjusted*_
Characteristic	(*n* = 572, 100.0%)	(*n* = 104, 18.2%)	(*n* = 441, 77.1%)	(*n* = 27, 4.7%)				
Samples	67442	7850	53793	5799				
Average samples per user	117.9	75.5	122.0	214.8				
Times reported tinnitus presence	52204 (77.4%)	5269 (67.1%)	43393 (80.7%)	3542 (61.1%)				
Device type					*χ*^2^ = 4.04 (2)	*V* = 0.08	0.133	0.292
iOS	301 (52.6%)	63 (60.6%)	222 (50.3%)	16 (59.3%)				
Android	271 (47.4%)	41 (39.4%)	219 (49.7%)	11 (40.7%)				
Gender					*χ*^2^ = 3.34 (2)	*V* = 0.08	0.188	0.345
Female	162 (28.3%)	37 (35.6%)	117 (26.5%)	8 (29.6%)				
Male	394 (68.9%)	65 (62.5%)	312 (70.7%)	17 (63.0%)				
Age	55.8 (13.1)	50.9 (13.2)	56.9 (12.9)	57.5 (11.7)	F = 8.80 (2)***	*η*^2^ = 0.03	<0.001	0.002
Age at onset of tinnitus	39.0 (14.6)	35.0 (15.0)	39.9 (14.5)	39.4 (13.8)	F = 4.66 (2)***	*η*^2^ = 0.02	0.010	0.054
Continent					*χ*^2^ = 12.61 (10)	V = 0.10	0.246	0.422
Europe	450 (78.7%)	86 (82.7%)	346 (78.5%)	18 (66.7%)				
North America	82 (14.3%)	11 (10.6%)	63 (14.3%)	8 (29.6%)				
South America	11 (1.9%)	4 (3.8%)	7 (1.6%)	0 (0.0%)				
Oceania	11 (1.9%)	2 (1.9%)	9 (2.0%)	0 (0.0%)				
Asia	10 (1.7%)	0 (0.0%)	9 (2.0%)	1 (3.7%)				
Africa	2 (0.3%)	0 (0.0%)	2 (0.5%)	0 (0.0%)				
miniTQ sum score	13.9 (5.7)	14.3 (5.3)	13.7 (5.8)	14.9 (5.0)	F = 1.04 (2)	*η*^2^ = 0.00	0.354	0.556
Is your tinnitus reduced by music or by certain types of environmental sounds such as the noise of a waterfall or the noise of running water when you are standing in the shower?	273 (47.7%)	61 (58.7%)	197 (44.7%)	15 (55.6%)	*χ*^2^ = 5.33 (2)*	*V* = 0.10	0.070	0.191
Does the presence of loud noise make your tinnitus worse?	164 (28.7%)	31 (29.8%)	125 (28.3%)	8 (29.6%)	*χ*^2^ = 0.45 (2)	*V* = 0.03	0.800	0.800
Do you have a problem tolerating sounds because they often seem much too loud? That is, do you often find too loud or hurtful sounds which other people around you find quite comfortable?	1.9 (1.1)	1.8 (1.1)	1.9 (1.1)	2.1 (0.9)	F = 0.48 (2)	*η*^2^ = 0.00	0.621	0.722
Do sounds cause you pain or physical discomfort?	162 (28.3%)	36 (34.6%)	118 (26.8%)	8 (29.6%)	*χ*^2^ = 0.84 (2)	*V* = 0.04	0.656	0.722
Do you suffer from headache?	117 (20.5%)	23 (22.1%)	91 (20.6%)	3 (11.1%)	*χ*^2^ = 1.40 (2)	*V* = 0.05	0.496	0.683
Do you think you have a hearing problem?	199 (34.8%)	33 (31.7%)	153 (34.7%)	13 (48.1%)	*χ*^2^ = 6.69 (2)**	*V* = 0.11	0.035	0.129

*T-* tinnitus reduction, *T0* no effect on tinnitus, *T+* tinnitus increase, *DoF* degrees of freedom, *p*_*adjusted*_ *p* values corrected for multiple comparisons using the Benjamini-Hochberg method, *p < 0.1, **p < 0.05, ***p < 0.01

Note that, when controlling for false discovery rate using the Benjamini-Hochberg method, only the difference in age remains significant. Therefore, the data should be considered as results of an exploratory analysis and consequently interpreted with appropriate caution to avoid false rejections of the null hypotheses.

## Discussion

To our knowledge, this is the first study in which subjective ecological momentary assessments were combined with sensor-based data about ambient sound in a large global cohort of tinnitus patients. Using this innovative technology, we were able to demonstrate that the effect of environmental sounds on tinnitus can be investigated under real-world conditions. We found an interindividually variable effect on subjective tinnitus loudness. According to the direction of the effect of environmental sounds, we could differentiate three groups: patients in which increasing environmental sound levels were associated with a reduction in subjective tinnitus loudness (T-), an increase in subjective tinnitus loudness (T+), or no change in tinnitus (T0). The T- group was much smaller than expected (18.2%), indicating that tinnitus masking occurs less likely with environmental and everyday sounds under real-world conditions as compared to experimental conditions with specifically selected sounds, where masking is typically observed in the majority of patients^28^. Exploratory analyses further revealed that the three groups (T-, T+, and T0) differed in age, age at onset of tinnitus (i.e., the age at which the patient first experienced tinnitus), and hearing problems, which suggests that the effect of sound on tinnitus is a relevant criterion for clinical subtyping.

The phenomenon that tinnitus can be masked by external sound stimuli has been known for a long time^9^. Investigations under clinical and laboratory conditions revealed that tinnitus can be masked in about 70% of patients^28^. It has been assumed that these patients may also experience some degree of masking from ambient environmental noise, which might tend to reduce the perceived severity of their tinnitus^28^. Sound stimulation has also been found to be the most commonly reported self-help strategy for tinnitus patients^29–31^. However, in controlled studies, no robust therapeutic effect of sound stimulation could be confirmed^13^. Until now, there has been no satisfactory explanation for this discrepancy between successful masking in the majority of tinnitus patients under laboratory conditions and the clinical lack of efficacy of sound therapy.

By combining ecological momentary assessment of tinnitus loudness with measurement of ambient sound level, we could demonstrate, that under real-world conditions the effect of environmental sound is highly variable: in only about 20% of the patients it is associated with a reduction of tinnitus, in about 75% of the patients it has no effect, and in about 5% it is associated with an increase of tinnitus. These data suggest that results from masking tinnitus with specialized sounds under controlled conditions cannot be readily extrapolated to masking tinnitus with ambient sounds under everyday conditions. Our findings are in line with the negative results of clinical trials investigating sound treatment^13^, but also with observations of pronounced tinnitus suppression by sound in selected patients^32^. Successful masking of tinnitus seems to depend strongly on individual factors, the type of masking sounds, and situational factors. Further studies investigating sound therapy should take these aspects into account.

The comparison of clinical characteristics across the three groups revealed significant differences. This, in turn, suggests that the effect of environmental sound on tinnitus perception represents a relevant criterion for patient subtyping.

It is noticeable that the groups differ in their age: the T- group is the youngest, followed by the T0 group and the T+ group, which is the eldest. This finding aligns well with findings from animal experiments that have shown an age-dependent reduction of inhibitory function in the central auditory pathways^33,34^. If inhibitory function in central auditory pathways is reduced in the elderly, it is conceivable that suppression of tinnitus by ambient sound can no longer be efficiently mediated. In addition, age-related differences in tinnitus perception and hearing thresholds were also observed in other studies^35,36^. Note that post-hoc tests indicated that the differences in age were only statistically significant between T0 and T-, but not between T0 and T+ and between T- and T+. This finding may be attributable to the small group size of T+. Moreover, the T- group is also the group in which the tinnitus occurred at a younger age (tinnitus onset). However, when adjusting for the current age, this effect is no longer significant. Thus, the finding of a younger age at onset in this group may be a pure consequence of the younger current age.

In addition, more hearing problems were reported in the T+ group than in the other groups. This may have an obvious reason: If ambient sounds do not reach the central auditory pathway because of cochlear damage, they cannot be heard and consequently cannot suppress tinnitus.

The proportion of women is highest in T-, followed by T+, and lowest in T0. This finding of a more pronounced positive effect of ambient sound in female tinnitus patients aligns very well with previous reports demonstrating better responses to sound stimulation in females than in males^37^. However, since this difference does not reach statistical significance, it should not be overinterpreted.

Given the international origin of the data in the analyzed sample, it is an interesting observation that there do not appear to be any systematic geographical differences in terms of the continental distribution of users between the identified groups. This indicates that the identified patient subtyping is possibly observable equally worldwide.

Interestingly, the observed effect of environmental sounds on tinnitus is only partially reflected by the subjective assessment of tinnitus maskability. When patients registered in the TYT app, they were asked: “Is your tinnitus reduced by music or by certain types of environmental sounds such as the noise of a waterfall or the noise of running water when you are standing in the shower?”. In line with the group classification, the proportion of patients answering “yes” to this question is the highest in T- and 14% higher than in T0, supporting the validity of our methodological approach. In contrast, however, still about 40% of patients in the T- group stated that their tinnitus is not maskable. Further research is needed to investigate the reasons for this discrepancy. Surprisingly, the proportion of subjective tinnitus maskability is similarly high in T+ as in T-. One possible explanation for this finding is the clinical experience that masking depends strongly on whether a sound is perceived as pleasant (e.g., natural sounds or music) or unpleasant (e.g., industrial or construction noise). In addition, studies suggest that one of the benefits of sound therapies is to provide a sense of control over tinnitus by controlling the sounds^38–41^, indicating that only desired and controllable sounds relieve tinnitus. Furthermore, studies have shown that masking effects depend on the acoustic properties of the masking sound^42^. Therefore, the type of ambient environmental sounds may confound its effects on tinnitus. This interaction should be investigated in further research.

The study has strengths and weaknesses. Strengths are the large sample size, the long duration of data collection, and the worldwide origin of the data of the investigated sample (see Final Dataset Description). A further strength is that the measured sounds were originally not intended to induce masking, which psychologically minimizes the anchor effect^43^. Moreover, participants were not aware that a relationship between ambient sound level and tinnitus severity will be investigated, which presumably reduced potentially confounding attentional and suggestive effects. This is an important difference in comparison to masking tests under laboratory conditions, where the expectation and attention is focused on a possible masking effect. Moreover, the users were well distributed between smartphone operating systems (Android and iOS), which we consider positive in light of previous results^44^. Interestingly, we also found no indication of a confounding effect of the smartphone operating system, as results were similar for both systems.

In interpreting the proportion of the three groups, some caution should be taken, as the number of measurements per participant varied across groups. In the T0 group, there were on average 122.0 measurements, in T- 75.5, and in T+ 214.8 respectively. We cannot exclude that for some participants of the T0 group, a higher or lower number of measurements would have led to a classification in T- or T+. In general, the use of other classification criteria may have resulted in a different subtyping.

Another limitation of this study is that no information was collected on the type of the environmental sounds. Since masking effects depend on the acoustic properties of the masking sound^42^, this information would be helpful for further investigation of masking effects.

Smartphone-based health data collection also has general weaknesses that must be considered in the interpretation of the results. As participation depended on an individual’s own motivation to use the app and to adhere to it, a selection bias must be assumed. About one in four registered users (26.4%) did not complete the comparatively comprehensive registration questionnaires and were therefore never able to use the EMA component of the TYT app. Furthermore, the majority (89.2%) of registered app users did not provide sufficient EMA data to be included in the analysis (see Data Preparation). This shows that the creation of sufficient adherence to a data collection tool is one of the most difficult tasks in the context of mHealth^45^. Moreover, we could not control how well users followed the instructions when they used the app. Even intentional manipulation cannot be excluded, as participants could theoretically enter fake data. In addition, some inaccuracy can be related to the sound measurements. The necessary run-up to compare the sound measurements in technical terms was costly, and it still cannot be ruled out that there are biases in the sound measurements^25,27^. Furthermore, we cannot detect, for example, whether the microphone was covered or touched while filling out the questionnaire^25^. To avoid such bias, we would have to remind the user before the completion of each questionnaire about the correct conditions for sound measurements, which in turn would create an attentional bias. In this study, this compromise was decided against in favor of the EMA questions, but it could have been worthwhile to improve the quality of the sound measurements and should be given more consideration in future studies.

Overall, however, the findings from our study are well compatible with existing knowledge, suggesting that the mentioned confounding factors do not play a decisive role. Thus, the innovative technological approach could contribute important information to gap the missing link between the high percentage of patients in which tinnitus can be masked under laboratory conditions and the relative limited effect of sound-based therapies.

## Methods

### Ethical approval declarations

The study was approved by the Ethics Committee of the University Clinic of Regensburg (ethical approval No. 15-101-0204). All users read and approved the written informed consent in the TYT app before participating in the study. The study was conducted according to the guidelines of the Declaration of Helsinki^46^.

### TrackYourTinnitus (TYT)

The TYT mobile platform uses EMA and MCS to monitor the variability of users’ individual tinnitus symptoms^23^. The platform has been in operation since 2014 and consists of (a) a website (https://www.trackyourtinnitus.org/) for sign-up and general information, (b) a central server backend for data storage, and (c) a mobile application available for both Android and iOS for data collection. The individual momentary tinnitus perception (e.g., tinnitus loudness and distress) is assessed by asking users to complete tinnitus EMA questionnaires at different semi-random or fixed times of the day (see TYT EMA-D Questionnaire). In addition, parallel with the completion of the daily questionnaire, the microphone of the device records environmental sound levels^24,25^. The detailed process of the TYT mobile application as well as the structure and insights of the collected dataset are described in previous work by the authors^26,47^. It could be demonstrated that regular use of the TYT app has no significant negative effects on perceived tinnitus loudness and tinnitus distress^23^.

### Recruitment

To recruit users, the TYT mobile application was advertised on the websites and social media pages of the Tinnitus Research Initiative, the COST Action TINNET, and the websites of the participating research groups^23^. As TYT is accessible free of charge via the Google Play Store and the Apple App Store, the only inclusion criterion for the study was the possession of a compatible smartphone. No other inclusion and exclusion criteria were applied. No additional instructions or incentives were given to users other than those in the TYT app itself.

### Clinical and demographic characteristics

Three “registration questionnaires” were completed once by users after they logged in to the TYT mobile application for the first time. The twelve-item version of the *Tinnitus Questionnaire (TQ)*, denoted *Mini-TQ*^48^, was used to assess the initial tinnitus-related psychological distress. In addition, demographic data of users (e.g., age and gender), as well as information on history and descriptive characteristics of their tinnitus or tinnitus-related conditions, were assessed using the *Tinnitus Sample Case History Questionnaire (TSCHQ)*^49^. The third questionnaire, which asked for the individually most disturbing tinnitus-related aspect in a single question, was not used in the present analysis.

### TYT EMA-D questionnaire

The *dynamic EMA questionnaire (EMA-D)* of the TYT mobile applications was used to assess the momentary tinnitus perception in everyday life. The questionnaire comprises eight questions to record tinnitus- and situation-specific variables^23^, which are presented in Table 3. The questionnaire can be completed any number of times at different times of the day^23,24^. Users are prompted to complete the questionnaire several times a day at either semi-random or fixed points in time via notifications on the mobile device. With the semi-random scheme, notifications are distributed semi-randomly within a time window per day according to an algorithm described by Pryss et al.^50^. With the fixed notification scheme, notifications are sent at fixed times specified by the user for each day of the week. The notification scheme (default: semi-random) and frequency (default: 3 per day) can be customized by the user at any time^24^.

**Table 3:** Questions of the EMA-D questionnaire in the TrackYourTinnitus (TYT) mobile application, along with their scale and the dimension that is measured^23^

#	Question	Scale	Range	Dimension
1	Did you perceive the tinnitus right now?	BS	{0,1}	perception
2	How loud is the tinnitus right now?	VAS	[0,1]	loudness
3	How stressful is the tinnitus right now?	VAS	[0,1]	distress
4	How is your mood right now?	VAS	[0,1]	valence
5	How is your arousal right now?	VAS	[0,1]	arousal
6	Do you feel stressed right now?	VAS	[0,1]	stress
7	How much did you concentrate on the things you are doing right now?	VAS	[0,1]	concentration
8	Do you feel < reported worst symptom > right now?	BS	{0, 1}	worst symptom

*BS* Binary Scale, *VAS* Visual Analogue Scale, Question #2 is used as the dependent variable tinnitus loudness (TL) in the present analysis.

### Environmental sound level

The average environmental sound level (SL) was recorded during the first 15 seconds while the user completed the EMA-D questionnaire using the device microphone^24,25^. The value is calculated by retrieving the average sound level via the respective system APIs on the mobile operating systems for each 500 ms period (2 Hz) and averaging these values in turn^25^. This procedure is subject to several limitations, namely heterogeneous hardware and software components between device models and operating systems, non-calibrated devices, lack of error detection and plausibility checks, lack of transparency of the user interface, and partially erroneous calculation, as discussed in previous work by the authors^25^. These limitations could only be identified retrospectively, meaning that the necessary software adjustments to address them could no longer be made. Note that for this reason, these values cannot be interpreted as sound pressure level (SPL) values or similar comparable units of measurement. To account for these limitations, the environmental sound levels were standardized prior to the statistical analysis, and the heterogeneity of these data was considered throughout the analysis. Users who contributed data from both iOS and Android devices were thereby each treated as two separate users. Also note that the measurement of sound involves the analysis of dimensions other than intensity, such as the frequency and temporal dimensions (e.g., duration, phase, and repetition rate) of acoustic signals^51^, which were not captured with the TYT mobile application. Furthermore, we did not record any information about the type and content of the environmental sound.

### Data preparation

**Fig. 3:**
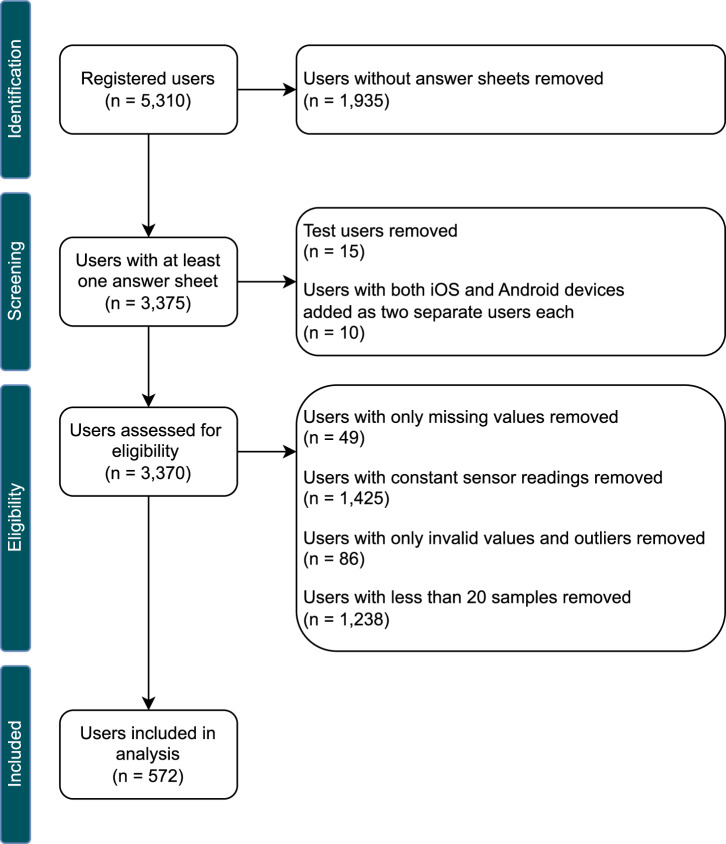
Data preparation. The steps used for data preparation and the resulting number of users included in the analysis per step.

The dataset for the analysis was extracted from the TYT database on 03 June 2024. It contains a total of *n* = 5, 310 registered users and 114, 578 EMA-D samples. In addition, the baseline (*n* = 4, 136) and demographic data (*n* = 3, 906) assessed with the respective questionnaires were extracted. Several steps were taken to prepare the dataset, as outlined in the following. The resulting numbers of users for each of these steps are shown in Fig. 3.

1. All users without any answer sheets were removed.
2. All samples from known test users (e.g., developers) were removed.
3. Users who contributed data from iOS and Android devices were treated each as two separate users. For this purpose, for each of these users, the samples collected through Android devices were assigned to a new pseudo user.
4. All duplicate samples were removed.
5. All samples with missing values for tinnitus loudness (TL) and/or environmental sound level (SL) were removed.
6. Users with only constant sensor readings were removed using a procedure similar to that described in previous work by the authors^27^.
7. SL values recorded on Android devices (*SL*_*Android*_) and iOS devices (*SL*_*iOS*_) were transformed to the same co-domain [0, 1]. Since *SL*_*Android*_ are stored as amplitude values, they can be divided by their maximum value to receive a relative value, as shown in Equation (1). The maximum amplitude value is given by the used Android MediaRecorder API, which returns absolute amplitude values in the range of [0, 32767]. Since *SL*_*iOS*_ are stored as relative decibel (dB) values, they can be transformed to a ratio (sound field quantity) with Equation (2).
8. For each user, samples with outliers for TL and/or SL were removed. Outliers were defined as values 3 or more standard deviations above/below the mean (z-score) of that user. In addition, samples with known invalid values (e.g., below a threshold) were removed.
9. All samples from users with less than 20 samples were removed. This step was performed to ensure that there were enough data points to investigate the correlation between TL and SL. Data from users with less than 20 samples were not considered sufficiently representative based on the experience of past analyses with the same dataset and pre-analyses with different thresholds.
10. Predictors with high skewness were transformed using Box-Cox^52^ or Yeo-Johnson^53^ transformations to approximate normality. Note that SL was transformed in this way because most of the values were close to zero due to the nature of sound levels.

1 $$S{L}_{ratio}=\frac{amplitude}{amplitud{e}_{ref}}=1{0}^{\frac{S{L}_{iOS}}{20}}\in [0,1]$$

2 $$S{L}_{ratio}=\frac{S{L}_{Android}}{amplitud{e}_{max}}\in [0,1],amplitud{e}_{max}=32767$$

### Final dataset description

**Fig. 4:**
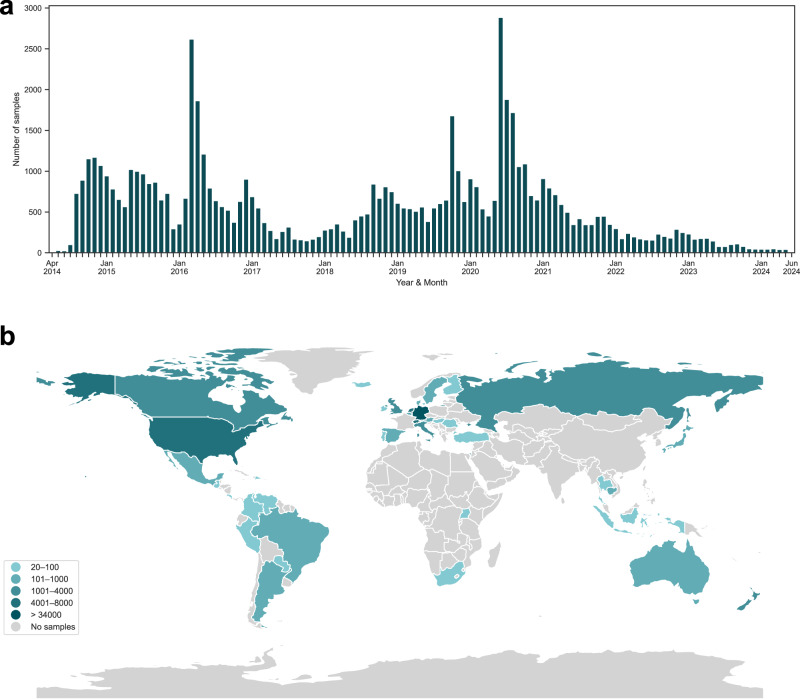
Distribution of samples. The number of EMA-D samples contributed (**a**) per year and month and (**b**) per country. Data were collected between April 2014 and June 2024.

The final dataset used for all analyses consists of a total of 67, 442 EMA-D samples from *n* = 572 unique users, along with the baseline and demographic data of these users. As shown in Fig. 4, these data were collected between April 2014 and June 2024 from users in 45 unique countries across the globe, with most samples collected in Germany (50.7%, *n* = 34, 173), followed by the United States (10.6%, *n* = 7, 142). Note that 1841 samples from 6 unique users could not be assigned a valid ISO 3166-1 country code. In terms of continents, most samples were collected in Europe (78.0%, *n* = 52, 573), followed by North America (14.0%, *n* = 9, 411), Oceania (2.0%, *n* = 1, 318), Asia (1.8%, *n* = 1, 199), South America (1.5%, *n* = 1, 012), and Africa (0.1%, *n* = 88). The distributions of the dependent variable tinnitus loudness (TL) and the main predictor variable environmental sound level (SL) can be found in Supplementary Figs. 1 and 2.

### Statistical analysis

All data were analyzed using Python 3.9.12 and R 4.4.0. Libraries used include pandas 1.4.2, NumPy 1.21.5, SciPy 1.7.3, scikit-learn 1.3.0, statsmodels 0.14.0, and pingouin 0.5.2. The package flexmix 2.3-19^54^ was used to perform the latent trajectory analysis. The libraries Matplotlib 3.7.2, seaborn 0.13.2, and geopandas 0.9.0 were used for data visualizations.

To identify subgroups of patients in terms of the relationship between environmental sound level (SL) and tinnitus loudness (TL), a latent trajectory analysis was performed using Latent Class Growth Analysis (LCGA) and Growth Mixture Modeling (GMM). We estimated the relationship between SL and TL using these person-centered approaches^55^ because our dataset consists of longitudinal, repeated measures of EMA tinnitus perceptions and sensor values for each participant. We used a structured and theory-driven approach to model specification and selection, following recommendations in the literature^56,57^ and considering the Guidelines for Reporting on Latent Trajectory Studies (GRoLTS)^58^. In this approach, we generally assumed a linear relationship between SL and TL, and used a forward modeling approach to determine the final model and the number of latent classes. We started with a one-class growth model and compared it to a two-, three-, and four-class model. First, we estimated LCGA models with homogeneous growth trajectories within each class (i.e., no within-class variance is assumed, with fixed intercept and slope per class). Second, we estimated GMM models incorporating within-class heterogeneity (in this analysis, with class-specific random intercepts). For both the LCGA and GMM models, the residual variances were fixed across latent classes. For the GMM models, the variance-covariance matrices were estimated freely (i.e., class-specific). All models were run 100 times with different random starting values, and only the solution with maximum likelihood was retained to avoid convergence to local maxima^58,59^. For the selection of the final model, we compared the models in terms of the Akaike information criterion, Bayesian information criterion^57^, log-likelihood, entropy, and trajectory plots with respect to theoretical assumptions to select the model with the best fit. A comparison of the tested models can be found in Supplementary Table 4. The 3-class GMM was selected as the final model.

We compared clinical and demographic characteristics between the three groups identified through the final growth model. For categorical variables, a *χ*^2^ test of independence was used. For continuous variables, a one-way ANOVA was performed, using *Tukey’s HSD* test as post-hoc test. Effect sizes were computed using *Cramer’s V* for categorical and *η*^2^ for continuous variables. Due to the exploratory nature of this analysis of descriptive statistics, *p*-values were not corrected for multiple comparisons. However, for the sake of completeness, corrected *p*-values are reported according to the method of Benjamini and Hochberg^60^.

## Supplementary information

Supplementary Information

## Data Availability

The datasets analyzed during the current study are not publicly available due to privacy reasons but are available from the corresponding author on reasonable request.

## Code availability

The underlying code for this study is not publicly available but may be made available to qualified researchers on reasonable request from the corresponding author.
