# Supplementary material for: Global 10 year ecological momentary assessment and mobile sensing study on tinnitus and environmental sounds
Source: NPJ Digit Med. 2025 Mar 13;8:162. doi: 10.1038/s41746-025-01551-z (PMC11906844; doi:10.1038/s41746-025-01551-z)
Supplement: Supplementary file 1 — Supplementary Information [file 41746_2025_1551_MOESM1_ESM.pdf]

Supplementary Information for: Global 10 Year Ecological Momentary Assessment and Mobile Sensing Study on Tinnitus and Environmental Sounds

Robin Kraft[1,2,3,4*], Berthold Langguth[5], Jorge Simoes[5,6], Manfred Reichert[3], Winfried Schlee[5,7†], Rüdiger Pryss[1,2*†]

[1]Institute of Medical Data Science, University Hospital Würzburg, Würzburg, 97080, Germany.
[2]Institute of Clinical Epidemiology and Biometry, University of Würzburg, Würzburg, 97080, Germany.
[3]Institute of Databases and Information Systems, Ulm University, Ulm, 89081, Germany.
[4]Department of Clinical Psychology and Psychotherapy, Ulm University, Ulm, 89081, Germany.
[5]Department of Psychiatry and Psychotherapy, University of Regensburg, Regensburg, 93053, Germany.
[6]Department of Psychology, Health and Technology, University of Twente, Enschede, 7522 NB, The Netherlands.
[7]Institute of Information and Process Management, Eastern Switzerland University of Applied Sciences, Rapperswil, 8640, Switzerland.

*Corresponding author(s). E-mail(s): robin.kraft@uni-wuerzburg.de; ruediger.pryss@uni-wuerzburg.de;
Contributing authors: berthold.langguth@medbo.de; j.pianosimoes@utwente.nl; manfred.reichert@uni-ulm.de; winfried.schlee@gmail.com;
[†]These authors contributed equally to this work.

1 Supplementary Figures

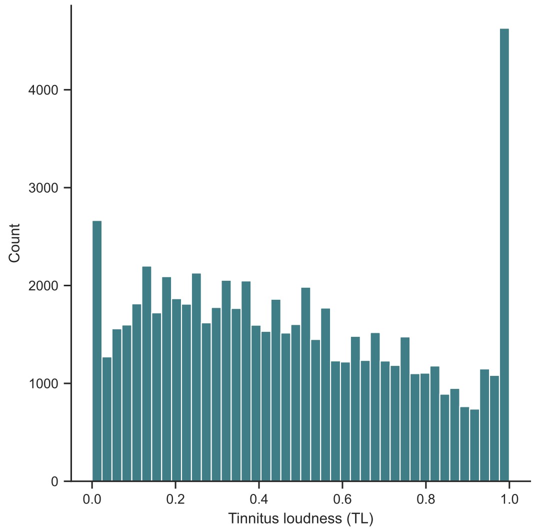

Supplementary Figure 1 Distribution of tinnitus loudness values. Histogram plot visualizing the distribution of the dependent variable tinnitus loudness (TL).

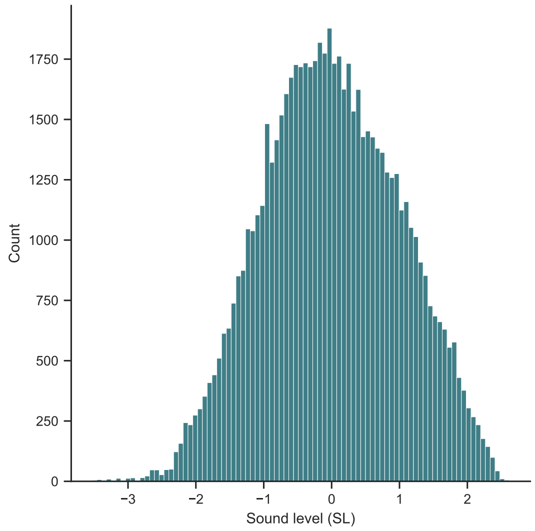

Supplementary Figure 2 Distribution of sound level values. Histogram plot visualizing the distribution of the main predictor variable environmental sound level (SL).

2 Supplementary Tables

Supplementary Table 1 Results of Tukey's HSD test between continuous variables of the T0 and T- group.

Characteristic	T0	T-	Mean Difference	p
age	56.900	50.919	5.981***	< 0.001
age at onset	39.929	34.970	4.959***	0.007
miniTQ sum score	13.677	14.343	-0.666	0.539

* $p < 0.1$, ** $p < 0.05$, *** $p < 0.01$

Supplementary Table 2 Results of Tukey's HSD test between continuous variables of the T0 and T+ group.

Characteristic	T0	T+	Mean Difference	p
age	56.900	57.542	0.641	0.970
age at onset	39.929	39.375	-0.554	0.982
miniTQ sum score	13.677	14.923	1.246	0.525

* $p < 0.1$, ** $p < 0.05$, *** $p < 0.01$

Supplementary Table 3 Results of Tukey's HSD test between continuous variables of the T- and T+ group.

Characteristic	T-	T+	Mean Difference	p
age	50.919	57.542	6.622*	0.064
age at onset	34.970	39.375	4.405	0.379
miniTQ sum score	14.343	14.923	0.58	0.889

* $p < 0.1$, ** $p < 0.05$, *** $p < 0.01$

Supplementary Table 4 Model comparison with fit indices for different LCGA and GMM models with 1–4 classes.

						Class sizes [1]			
**Model**	**#Classes**	**LL**	**AIC**	**BIC**	**Entropy**	1	2	3	4
LCGA	1	-13543.34	27092.69	27120.05	-	572			
	2	6478.98	-12945.97	-12891.25	.985	370	202		
	3	13556.63	-27095.25	-27013.18	.977	241	256	75	
	4	17265.46	-34506.92	-34397.49	.974	150	219	49	154
GMM	1	21132.94	-42257.88	-42221.40	-	572			
	2	21539.66	-43063.32	-42990.37	.720	460	112		
	3	21654.54	-43285.08	-43175.66	.746	104	441	27	
	4	21664.34	-43296.68	-43150.78	.536	27	78	238	229

LCGA: Latent Class Growth Analysis, GMM: Growth Mixture Modeling, LL: Log-likelihood, AIC: Akaike information criterion, BIC: Bayesian information criterion.

[1] Sample size per class based on most likely class membership.
